# A Unified Theoretical Framework for Cognitive Sequencing

**DOI:** 10.3389/fpsyg.2016.01821

**Published:** 2016-11-18

**Authors:** Tejas Savalia, Anuj Shukla, Raju S. Bapi

**Affiliations:** ^1^Cognitive Science Lab, International Institute of Information TechnologyHyderabad, India; ^2^School of Computer and Information Sciences, University of HyderabadHyderabad, India

**Keywords:** implicit sequence learning, explicit sequence knowledge, habitual and goal-directed behavior, model-free vs. model-based learning, hierarchical reinforcement learning

## Abstract

The capacity to sequence information is central to human performance. Sequencing ability forms the foundation stone for higher order cognition related to language and goal-directed planning. Information related to the order of items, their timing, chunking and hierarchical organization are important aspects in sequencing. Past research on sequencing has emphasized two distinct and independent dichotomies: implicit vs. explicit and goal-directed vs. habits. We propose a theoretical framework unifying these two streams. Our proposal relies on brain's ability to implicitly extract statistical regularities from the stream of stimuli and with attentional engagement organizing sequences explicitly and hierarchically. Similarly, sequences that need to be assembled purposively to accomplish a goal require engagement of attentional processes. With repetition, these goal-directed plans become habits with concomitant disengagement of attention. Thus, attention and awareness play a crucial role in the implicit-to-explicit transition as well as in how goal-directed plans become automatic habits. Cortico-subcortical loops basal ganglia-frontal cortex and hippocampus-frontal cortex loops mediate the transition process. We show how the computational principles of model-free and model-based learning paradigms, along with a pivotal role for attention and awareness, offer a unifying framework for these two dichotomies. Based on this framework, we make testable predictions related to the potential influence of response-to-stimulus interval (RSI) on developing awareness in implicit learning tasks.

## 1. Introduction

*Cognitive Sequencing* can be viewed as the ability to perceive, represent and execute a set of actions that follow a particular order. This ability underlies vast areas of human activity including, statistical learning, artificial grammar learning, skill learning, planning, problem solving, speech and language. Many human behaviors ranging from walking to complex decision making in chess involve sequence processing (Clegg et al., 1998; Bapi et al., 2005). Such sequencing ability often involves processing repeating patterns—learning while perceiving the recurrent stimuli or actions and executing accordingly. Sequencing behavior has been studied in two contrasting paradigms: *goal-directed* and *habitual* or under the popular rubric of *response-outcome* (R-O) and *stimulus-response* (S-R) behavior. A similar dichotomy exists on the computational side under the alias of *model-based* vs. *model-free* mechanisms. The model-based vs. model-free computational paradigm has proved vital in designing algorithms for planning and learning in various intelligent system architectures—leading to the proposal of their involvement in human behavior as well. In this article, we use another dichotomy on the learning side: *implicit* vs. *explicit* along with a pivotal role for attention and awareness to connect these dichotomies and suggest a unified theoretical framework targeted toward sequence acquisition and execution. In the following, the three dichotomies will be described along with a summary of the known neural bases of these.

### 1.1. Habitual vs. goal-directed behavior

Existence of a combination of habitual and goal-directed behaviors is shown in empirical studies on rats and humans. In the experiments to study these behaviors two phenomena have been used to differentiate: *outcome devaluation*—sensitivity to devaluation of the goal and *contingency degradation*—sensitivity to an omission schedule. Outcome devaluation is achieved by satiating the rats on the rewarding goal; making the reward less appealing whereas contingency degradation is achieved by omitting a stimulus within a sequence of stimuli leading to the goal. Results demonstrate that overtrained rats and humans seem to be insensitive to both the phenomena. That is, even though the outcome of following a path is devalued or a stimulus in the sequence is omitted, habits lead rats to follow the same path, thus relating overtraining to habitual or stimulus-response (S-R) kind of control (Adams and Dickinson, 1981; Killcross and Coutureau, 2003). On the other hand, moderately trained rats have had little or no difficulty adapting to the new schedule relating this behavior to a goal-directed or response-outcome (R-O) kind of control (Dickinson, 1985; Balleine and Dickinson, 1998; Tricomi et al., 2004; Balleine and O'Doherty, 2010; Dolan and Dayan, 2013). Based on this proposal of two contrasting mechanisms, quite a few notable neuroimaging studies have attempted establishing the neural substrate related to the two modes of control. fMRI studies related to outcome devaluation point to two sub areas of the ventromedial prefrontal cortex (vmPFC)—medial orbito-frontal cortex (OFC) and medial prefrontal cortex (PFC) as well as one of the target areas of the vmPFC structures in the human striatum, namely, the anterior caudate nucleus, to be involved in goal-directed actions (Valentin et al., 2007; Balleine and O'Doherty, 2010). Studies aimed at finding the neural substrate for habitual behavior suggest an involvement of the subcortical structure, dorsolateral striatum (Hikosaka et al., 1999; Yin and Knowlton, 2006; Tricomi et al., 2009).

### 1.2. Model-free vs. model-based paradigm

In order to understand the learning process in goal-directed and habitual paradigms, two contrasting computational theories have been proposed. Goal-directed learning and control processes have been related to a *model-based* system (Doya et al., 2002; Khamassi and Humphries, 2012) whereas the habitual paradigm to a *model-free* system (Dolan and Dayan, 2013). Typically, a goal-directed system uses its view of the environment to evaluate its current state and possible future states, selecting an action that yields the highest reward. A model-based mechanism conceives this as building a search tree leading toward goal states. Such a system can be viewed as using the past experiences to understand the environment and using this view of the environment to predict the future.

In contrast, a habitual behavior can be viewed as a repetition of action sequences based on past experience. Acquisition of a habit can be viewed as learning a skill—correcting the residual error in the action sequence leading to the goal. Typically skills are acquired over time, are sensitive to the effectors used for acquisition (Bapi et al., 2000) and there seems to be a network of brain areas involved at various stages of skill learning (Hikosaka et al., 1999). A model-free system conceives the residual error as a sort of prediction error called the *temporal difference* (TD) error (Sutton, 1988). Dopamine has been noted to be a key neurotransmitter involved in encoding the prediction error and thus leading to the view that dopamine plays a crucial role in habit learning (Schultz et al., 1997). The influence of dopamine is in no way limited to habitual behaviors. Role of dopamine in functions mediated by the prefrontal cortex such as working memory, along with the observation that there are wide projections of dopamine to both the caudate and the putamen and studies manipulating dopamine levels in the prefrontal cortex affecting goal-directed behavior indicate its involvement in the goal directed mechanism as well (see Dolan and Dayan, 2013 for a review).

Further work has been directed at establishing a connection between the dichotomies—the behavioral dichotomy of goal-directed vs. habitual and the computational dichotomy of model-based vs. model-free. For example, Daw et al. (2005) suggested an uncertainty based competition between the two behaviors—the computationally simpler process at any point acting as a driver process. Another interesting aspect of such a combination comes from the *hierarchical reinforcement learning* (HRL) framework (Sutton and Barto, 1998; Sutton et al., 1999; Botvinick et al., 2009; Botvinick, 2012). An important aspect of acquisition of sequences is the formation of chunks among the sequence of stimuli. The striatum has been emphasized to be involved in the chunking process, the chunks then selected and scaled by the output circuits of the basal ganglia (Graybiel, 1998).

### 1.3. Implicit and explicit learning

For the past three decades, there has been significant interest in sequence learning. A large body of experimental evidence suggests that there is an important distinction between *implicit* and *explicit learning* (see for example Howard and Howard, 1992; Shea et al., 2001 studies). Howard and Howard (1992) used a typical serial reaction time (SRT) task (Nissen and Bullemer, 1987), where the stimuli appeared in one of the four locations on the screen, and a key was associated with each location. The participants' job was to press the key as soon as possible. The stimuli were presented in a sequential order. With practice, participants exhibited response time benefits by responding faster for the sequence. However, their performance dropped to chance-level when they were asked to predict the next possible location of the stimulus suggesting that the participants might have learned the sequence of responses in an implicit manner and thus can not predict the next move explicitly. Another example is the study by Shea et al. (2001), where participants were given a standing platform task and were asked to mimic the movements of the line presented on a screen. The order of the stimuli was designed in such a way that the middle segment was always fixed whereas the first and the last segments varied but participants were not told about the stimulus order. It was found that the performance of the middle segment improves over time. During the recognition phase participants fail to recognize the repeated segment, pointing to the possibility that they may have acquired this via implicit learning. A recent study done with a variant of SRT task called oculomotor serial response time (SORT) task also suggests that motor sequence can be learned implicitly in the saccadic system (Kinder et al., 2008) as well and does not pose attentional demands when the SORT task is performed under dual-task condition (Shukla, 2012). Another way of differentiating implicit vs. explicit learning would be to see whether an explicit instruction was given about the presence of a sequence prior to the task. An instruction specifying the presence of a sequence in the task would, in turn, drive attentional learning. Without such explicit prior knowledge, however, it may take more number of trials for the subjects to become aware of the presence of a sequence—requiring them to engage their attention toward the sequence, turning the concomitant learning and execution explicit. Apart from these studies, there is a large body of clinical literature which confirms the distinction of implicit and explicit learning. Most of the clinical evidence comes from the *artificial grammar learning* (AGL) paradigm, where patients learned to decide whether the string of letters followed grammatical rules or not. Healthy participants were found to learn to categorize grammatical and ungrammatical strings without being able to verbalize the grammatical rules. Evidence from amnesic patients points toward implicit learning being intact in patients even though their explicit learning was severely impaired (Knowlton et al., 1992; Knowlton and Squire, 1996; Gooding et al., 2000; Van Tilborg et al., 2011). Willingham et al. (2002) suggested that activation in the left prefrontal cortex was a prerequisite for such awareness along with activation of the anterior striatum (Jueptner et al., 1997a,b). Results of the positron emission tomography (PET) study of Grafton et al. (1995) when participants performed a motor sequence learning task under implicit or explicit learning task conditions suggest that the motor cortex and supplementary motor areas were activated for implicit learning whereas the right premotor cortex, the dorsolateral cingulate, anterior cingulate, parietal cortex and also the lateral temporal cortex were associated with explicit procedural memories (Gazzaniga, 2004; Destrebecqz et al., 2005).

It has been established that the brain areas involved in working memory and attentional processing are more active during explicit learning as compared to implicit learning. Further, the findings of functional magnetic resonance imaging (fMRI) studies suggest that the prefrontal and anterior cingulate cortex and early visual areas are involved in both implicit and explicit learning (Aizenstein et al., 2004). However, there is a greater prefrontal activation in case of explicit processing than implicit which is consistent with the findings from attention literature suggesting that prefrontal activation is associated with controlled and effortful processing (Aizenstein et al., 2004). However, the neural bases of implicit and explicit learning are still inconclusive. For example, Schendan et al. (2003) used fMRI to differentiate brain activation involved in implicit and explicit processing. Their finding suggests that the same brain areas are activated in both types of processing. More specifically, the medial temporal lobe (MTL) is involved in both implicit and explicit learning when a higher order sequence was given to the participants. Furthermore, Pammi et al. (2012) observed a shift in fronto-parietal activation from anterior to posterior areas during complex sequence learning, indicating a shift in control of sequence reproduction with help of a chunking mechanism.

In this section we discussed the three dichotomies that have stayed mostly distinct in the literature. While there have been many significant attempts at combining goal-directed behavior with model-based mechanism and habitual behavior with model-Free mechanism, we attempt to add the third implicit vs. explicit dichotomy to devise a unifying framework explaining both learning and execution.

## 2. Computational equivalents

In this section we present how explicit learning and goal directed behavior can be related to a model-based mechanism whereas implicit learning and habitual behavior can be related to a model-free system. Indeed, there have been previous such attempts at bringing together the contrasting paradigms (Doya et al., 2002; Daw et al., 2005; Dezfouli and Balleine, 2012; Dolan and Dayan, 2013; Dezfouli et al., 2014; Cushman and Morris, 2015).

### 2.1. Goal directed behavior as model-based mechanism

A goal directed behavior can be viewed as keeping the end-point (goal) in mind and selecting the ensuing actions accordingly. This kind of learning and control can be explained by a simple markov decision process (MDP) framework. Typically, an agent estimates its environment and calculates the value of its current state and possible future states. This estimation can be described by the Bellman equations:

(1) $$\begin{array}{l} V\left(s_{n}\right)=\sum T\left(s_{n},a,s_{n+1}\right)\left[R\left(s_{n},a,s_{n+1}\right)+\gamma V\left(s_{n+1}\right)\right] \end{array}$$

Here, *T*(*s*_*n*_, *a, s*_*n*+1_) is the transition probability: the probability of the agent landing in state *s*_*n*+1_ if it takes an action *a* from state *s*_*n*_. *R*(*s*_*n*_, *a, s*_*n*+1_) denotes the reward the agent gets on taking an action *a* from state *s*_*n*_ and lands in state *s*_*n*+1_, γ the discount factor enabling the agent to select higher-reward-giving actions first. Such a system can be viewed as building a search tree and looking forward into the future, estimating values of the future states and selecting the maximum valued one (denoted as $V^{*}\left(s_{n}\right)$).

(2) $$\begin{array}{l} V^{*}\left(s_{n}\right)=\underset{a}{\max}\sum T\left(s_{n},a,s_{n+1}\right)\left[R\left(s_{n},a,s_{n+1}\right)+\gamma V\left(s_{n+1}\right)\right] \end{array}$$

This kind of behavior requires the agent to know the transition probabilities along with the reward function. While these values are not directly available in the environment, the agent builds these gradually over time while interacting with the environment—learning the transition probabilities iteratively. These values, in effect, form a model of the environment, hence deriving its name. Rewards in all the future states are estimated by propagating back the rewards from the final goal state. The transition probabilities can be initially equal for all actions and subsequently getting refined with experience.

### 2.2. Habitual behaviors as model-free system

Habitual behavior can be viewed as a typical S-R behavior, where the end-goal does not influence the current action selection directly. Instead, previous experiences of being in a particular state are cached (Daw et al., 2005). This can be conveyed by a model-free system through the well established temporal difference (TD) learning. TD learning follows the following update rules.

(3) $$\begin{array}{r} p_{k}=R\left(s_{n},a,s_{n+1}\right)+\gamma V\left(s_{n+1}\right); \end{array}$$

(4) $$\begin{array}{r} V\left(s_{n}\right)=\left(1-\alpha \right)V\left(s_{n}\right)+\left(\alpha \right)p_{k}\\ V\left(s_{n}\right)=V\left(s_{n}\right)+\alpha \left(V\left(s_{n}\right)-p_{k}\right) \end{array}$$

Here α is the learning rate. *p*_*k*_ encodes a sample evaluation of state *s*_*n*_ when the agent enters state *s*_*n*_ for the *k*th time in the form of a sum of two terms—the first term indicating reward the agent would receive from the current state and the second term computing discounted value of the next state *s*_*n*+1_ that it would enter, the value being returned is the agent's version of the value function *V*(·). The last term of Equation (4) refers to the prediction error signal. The definition of terms such as *R*(·) and *V*(·) are as defined earlier in Bellman equations. This system can be viewed as looking into the past—making a small adjustment to optimize performance and taking the next action. There is no explicit model of the system, the agent learns on-line—learning while performing.

Our proposed architecture attempts a combination of the two contrasting paradigms. We suggest that implicit learning and control can be viewed in a similar way as habitual behavior and in turn both can be modeled using a model-free computational system. Similarly, explicit learning and control seem to have similar requirements as goal-directed behavior and in turn both can be understood as using a model-based computational system. We aim to exploit the hierarchical reinforcement learning architecture and chunking phenomenon to propose how these contrasting dichotomies can be combined into a unified framework in the next section.

## 3. Unified theoretical framework

In a model-based mechanism—searching in a tree of possible states—as one looks further ahead into the future, the search tree starts expanding exponentially, making such a search computationally infeasible. Whereas in case of a model-free mechanism, the system has to be in the exact same state as it was before to enable an update in its policy. To make such an update account for something substantial, there have to be enough samples of a particular state which might take a larger number of trials exploring the entire state space. The respective inefficiency of the individual systems (Keramati et al., 2011) and the evidence of existence of both as part of a continuum allow us to formulate a hybrid scheme combining both the computational mechanisms to explain sequence acquisition and execution in the brain.

In an attempt to formulate a unifying computational theory, we add the learning factor—implicit learning conceived as model-free learning whereas explicit learning conceived as model-based. One such idea in computational theories that suits our needs is the hierarchical reinforcement learning (HRL) framework (Sutton and Barto, 1998; Sutton et al., 1999). The HRL framework gives an additional power to the agent: the ability to select an “option” along with a primitive action for the next step. An option is a set of sequential actions (a motor program) leading to a subgoal. The agent is allowed to have separate policies for different options—on selection of an option, the agent follows that option's policy; irrespective of what the “external” policy for the primitive actions is.

We propose that learning within an option—the policy of the primitive actions within an option occurs in a model-free way. The most granular set of actions a human performs are learned by a habitual mechanism and implicitly. As one moves to learning of a less granular set of actions the roles start to change—a habitual model-free learning gradually transforms to a goal-directed one. At some point, one becomes aware of the recurring patterns being experienced and the attentional processes thereafter enable a shift from implicit state to explicit learning. Indeed, in the serial reaction time studies, it has been observed that as the subjects became aware of the recurring pattern or sequence, their learning might have moved from implicit to explicit state. We attribute this conversion to explicit learning to the formation of *explicit motor programs* or *chunks*. Chunks are formed when the subject becomes aware of the sequential pattern and implicit, model-free learning then turns into an explicit and model-based learning process. One interesting theory that can be used to explain the chunking process is the *average reward Reinforcement Learning* model (Dezfouli and Balleine, 2012).

**Figure 1:**
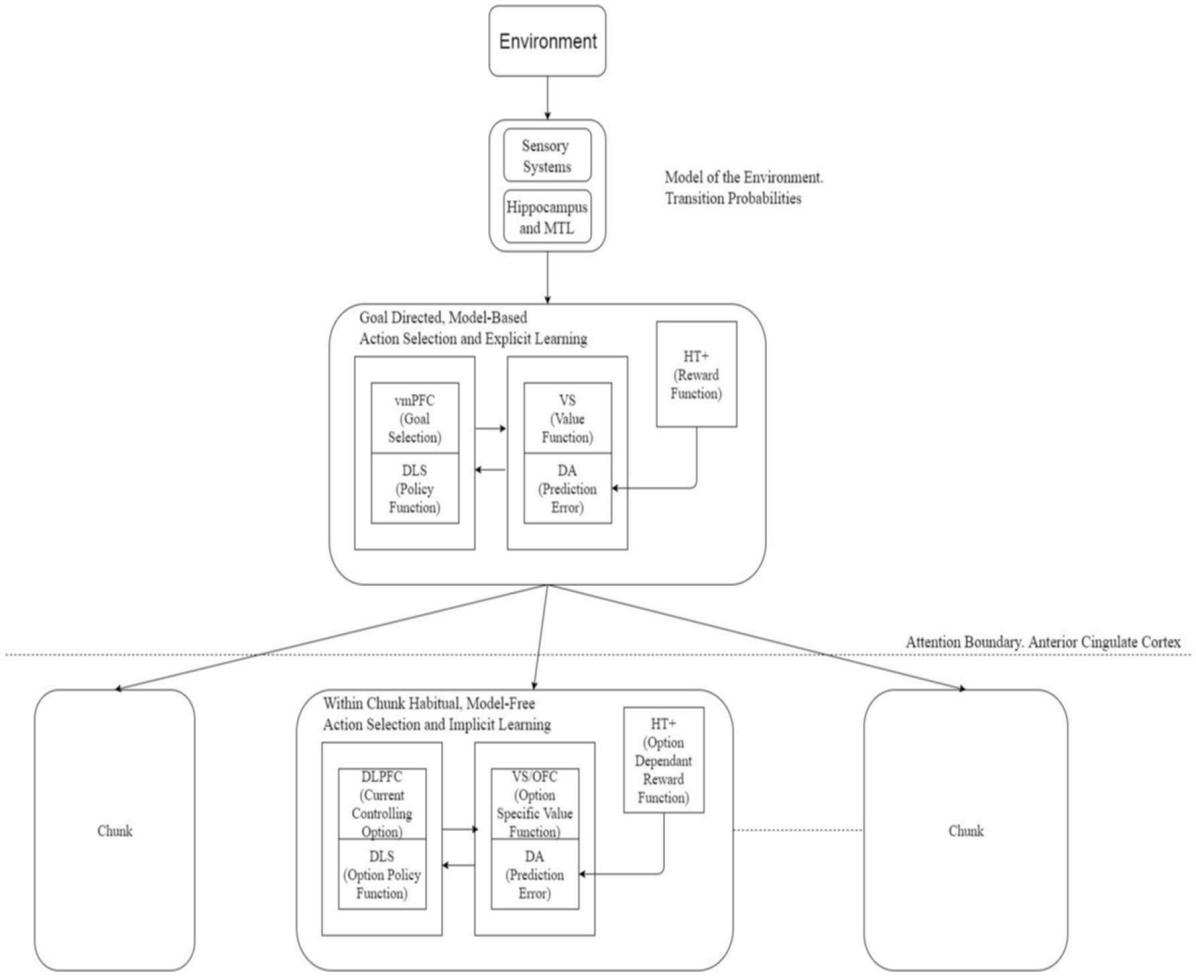
**Actor critic architecture for learning and execution**. Input from the environment passes to the goal-directed mechanism to select a chunk. Action selection at the upper-level is enabled by engagement of attention in a goal-directed, model-based manner whereas at a lower level (without attentional engagement) this process implements habitual and model-free system. Action selection within a chunk occurs on a habitual, model-free basis. Neural correlates of various components of this framework are suggested here. Based on Botvinick et al. (2009). vmPFC, Ventromedial PreFrontal Cortex; DLS, DorsoLateral Striatum; VS, Ventral Striatum; DA, Dopaminergic error signal; HT+, Hypothalamus; DLPFC, DorsoLateral PreFrontal Cortex; OFC, OrbitoFrontal Cortex; MTL, Medial Temporal Lobe.

As depicted in Figure 1, a similar analogy can be applied in control or performance of sequences with a change in direction in the process described above. The most abstract, top level goals are executed explicitly in a goal-directed way using model-based mechanism, the goal directed mechanisms gradually relinquishing control as the type of actions proceed downward in the hierarchy. At the finest chunk-level, subject loses awareness of the most primitive actions executed and those are then executed entirely in a habitual, model-free, implicit manner.

In neural terms, the ventromedial prefrontal cortex (vmPFC) along with the caudate nuclei may be involved in the goal-directed part, the dorsolateral striatum and dorsolateral prefrontal cortex (DLPFC) may be engaged in the habitual part—dopamine providing the prediction error signal while the anterior regions of the striatum and left prefrontal and medial frontal cortex playing a role in attentional processes. Gershman and Niv (2010) suggested a role for the hippocampus in task structure estimation which could be extended to estimating the world model and hence the transition probabilities required for the model-based system. Neural correlates for the options framework are detailed in Figure 1.

In this section we presented our unifying framework for combining the three dichotomies. In the subsequent section we attempt to specify the roles of response-to-stimulus interval (RSI) [or more generally, inter-stimulus interval (ISI)] and prior information along with the pivotal role of attention in switching between the two contrasting mechanisms in the explicit vs. implicit and goal-directed vs. habitual dichotomies.

## 4. Role of response-to-stimulus-interval (RSI) and prior information in the unified framework

A model-based search leading to explicit learning is typically slower—subject is required to deliberate over possible choices leading to the goal. In contrast, subject does not need to think while performing an action habitually or learning implicitly—a model-free mechanism does not deliberate, it performs an action based on an already available “cache” of previous experiences and updates the cache as it proceeds further. Based on this, we propose that *response-to-stimulus interval* (RSI) [or more generally, inter-stimulus interval (ISI)] plays a key role in serial reaction time (SRT) experiments. Larger RSIs allow the subject enough time to form a model of the system, deliberate over the actions and hence this kind of learning and control corresponds to a model-based (explicit) system. On the other hand smaller RSIs do not allow the subject to form an explicit model and as is well known from the literature of serial reaction time experiments, subjects do remain sensitive to (implicitly acquire) the underlying sequential regularities (Robertson, 2007). This sort of implicit learning can be explained with temporal difference (TD) learning, where the error signal leads to an adjustment in action selection keeping the general habitual control the same.

**Figure 2:**
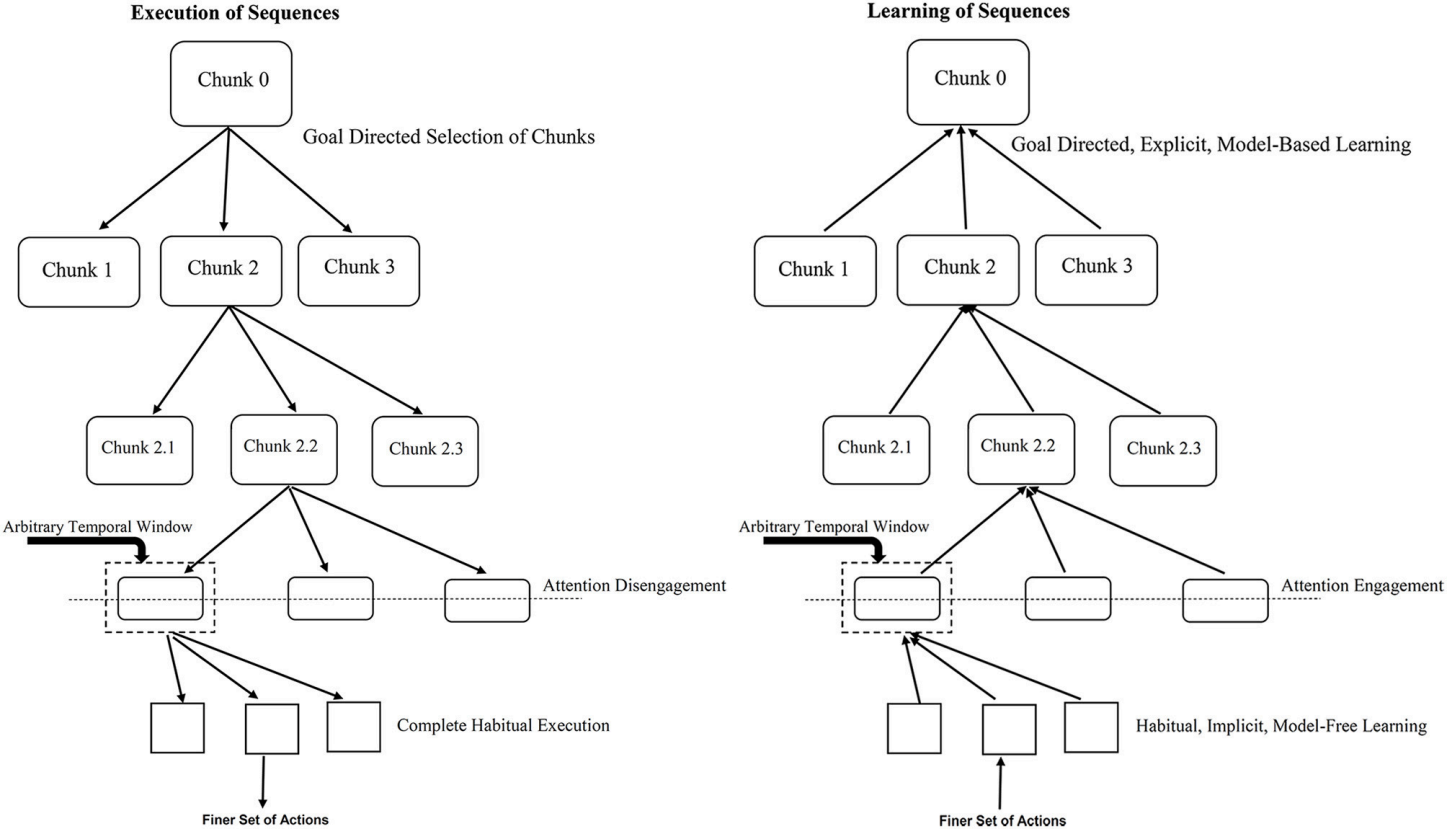
**Role of temporal window in engagement/disengagement of attention during learning and execution**. The left panel refers to sequence execution (performance) where the flow is from top-to-bottom, attention gets gradually disengaged as you go down the hierarchy. The right panel shows the acquisition (learning) of sequences where the flow is from bottom-to-top, attention gets gradually engaged as you go up the hierarchy. The temporal window determines when to switch between the two mechanisms. For example, for an action worth 1 unit of time with the temporal window size of 5 units with RSI of 3 units; a two-action chunk would lead to attention engagement/disengagement. Lesser RSI would require more number of actions chunked together to engage/disengage attention toward the underlying task.

Further, knowledge (prior information) about the existence of sequential regularities in the SRT task leads to the learning and control being explicit and model-based. This can be said to engage attentional processes in our proposal. With attentional engagement, habitual control ceases to exercise control over behavior. While we propose attention-mediated arbitration between model-based and model-free systems, Lee et al. (2014) suggest that such mediation is driven by estimates of reliability of prediction of the two models. Emerging awareness of the presence of a sequence plays a similar role in mediating learning as explicit attentional processes. The complete architecture is depicted in Figure 2.

Our proposal relies heavily on the *hierarchical chunking* mechanism and engagement or disengagement of attention to the underlying repeating pattern or sequence. While learning which begins implicitly and in a model-free manner, eventually as the formation of chunks proceeds up the hierarchy, at some point, the size of chunk—defined in terms of the time it takes to execute the set of actions within the chunk, crosses a threshold thus engaging the attentional resources of the subject. At this point explicit model-based learning starts taking control. Similarly, during control (or execution) of a sequence, the top most selection of chunks happens via a goal-directed, model-based mechanism, on proceeding down the chunk hierarchy after the point of crossing some chunk size threshold, the subject no longer pays attention to the execution—it goes on in a habitual, model-free manner. Learning or execution of a set of actions within a chunk proceeds in a habitual, model-free fashion, – which at “attentive” level in the hierarchy can be explained by a habitual control of goal selection as suggested by Cushman and Morris (2015).

Attention engagement or disengagement occurs when the chunk size is equivalent to a certain *temporal window*. Such a temporal window includes the RSI for a typical SRT task. For instance, larger RSIs need fewer physical actions to reach the threshold size of the temporal window during bottom-up learning and hence cause attentional engagement toward the underlying sequential pattern sooner than in case of a trial with smaller RSIs. Based on this proposal, it will be interesting to empirically investigate the impact of varying the size of temporal window and studying resultant influence on the awareness levels of the presence of an underlying sequence in the standard SRT task. According to our proposal, implicit (associative) learning in the lower-level of the hierarchy proceeds without engagement of attention. Further we propose that as the response-stimulus interval (RSI) increases the width of the temporal window available for integration of information related to the previous response and the subsequent stimulus increases. Thus, increasing the temporal window allows deliberative and reflective (analytical) processes to kick in, enabling a transition to explict (awareness-driven) top-down mechanisms. This prediction can be verified experimentally and seems to be supported by preliminary evidence from the work of Cleermans et al. (see Cleeremans 2014).

Such a hierarchical chunking mechanism for behavior generation has been suggested by Albus (1991), albeit from intelligent control architecture perspective. According to Albus (1991), a hierarchy producing intelligent behaviors comprises seven levels covering at the lowest level, the finest reflex actions, and spanning all the way up to long term planning of goals. At each higher level, the goals expand in scope and planning horizons expand in space and time. In neural terms, the motor neuron, spinal motor centers and cerebellum, the red nucleus, substantia nigra and the primary motor cortex, basal ganglia and prefrontal cortex and finally the temporal, limbic and frontal cortical areas get involved in increasing order of the hierarchy.

## 5. Comparison with other dual system theories

Many dual system theories related to goal-directed vs. habitual behavior or implicit vs. explicit learning have been proposed in the recent past. For example, Keele et al. (2003) suggest a dual system where implicit learning is typically limited to a single dimensional or a unimodal system whereas explicit learning involves inputs from other dimensions as well. Our model incorporates this duality in a different sense and does not distinguish the dichotomy between different modalities. Inputs from multiple modalities are treated as actions in an abstract sense and when a bunch of such actions crosses the threshold (acquisition or execution time), this would lead to attentional modulation (engagement in the case of acquisition or disengagement in the case of performance). A similar idea has been discussed by Cleeremans (2006) who suggested that a representation obtained from exposure to a sequence may become explicit when strength of activation reaches a critical level. Formation of the chunks is, however, assumed to be driven by bottom-up, unconscious processes. These chunks become available later for conscious processing (Perruchet and Pacton, 2006). We concur with the suggestions of Keele et al. (2003) on the neural correlates of implicit and explicit learning; learning in the dorsal system being implicit whereas that in the ventral system may be related to explicit or implicit modes. However, we emphasize that the ventral system—when learning is not characterized as a uni- or multi-dimensional dichotomy—would be more related to explicit learning. Daltrozzo and Conway (2014) discuss three levels of processing: an abstract level storage for higher level goals, followed by an intermediate level encoding of the actions required to reach the goal and a low level acquiring highly specific information related to the exact stimulus and associated final motor action (Clegg et al., 1998). Our model reflects such a hierarchy by breaking down the actions into a finer set of sub-actions—where the top most abstract actions or goals are decided by a goal-directed, model-based system whereas the more concrete actions are executed by a habitual, model-free system. Walk and Conway (2011) suggest a cascading account where two mechanisms interact with each other in a hierarchical manner—concrete information being encoded in a modality specific format followed by encoding of more domain-general representations. We incorporate such an interleaving phenomenon by suggesting that the actions within a chunk are carried out in a habitual, attention-free manner; the selection of such a chunk being goal-directed and attention-mediated. Thiessen et al. (2013) discuss a dual system involving an extraction and integration framework for general statistical learning. The extraction framework is implicated in conditional statistical learning—formation of chunks or associations between events occurring together. On the other hand, the integration framework is implicated in distributional statistical learning—generalization of the task at hand. We can relate the extraction framework to the implicit, habitual process and the integration framework to a goal-directed mechanism that involves creation of the model of environment using information from potentially multiple sources. Batterink et al. (2015) present evidence suggesting that though there does not seem to be an explicit recognition of statistical regularities, the reaction time task, which is deemed 50% more sensitive to statistical learning, suggests that there is in fact some statistical structure of the presented stimuli learned implicitly. Our framework agrees with the conclusion that implicit and explicit statistical learning occur in parallel, attention deciding the driver process. A similar account has been suggested by Dale et al. (2012) who state that the system initially learns a readiness response to the task in an associative fashion mapping the stimulus to a response and then undergoes a rapid transition into a “predictive mode” as task regularities are decoded. Reber (2013) suggests a role for the hippocampal-medial temporal lobe (MTL) loop in explicit memory whereas implicit memory is said to be distributed in the cortical areas. However, evidence from studies with Parkinsons patients suggests an important role for the basal ganglia in acquiring such implicit knowledge. We posit a similar role for the basal ganglia and corticostriatal loops in implicit learning; the knowledge that follows this learning may be stored throughout the cortex while keeping the role of MTL and hippocampus intact.

## 6. Conclusion and future work

Sequencing is a fundamental ability that underlies a host of human capacities, especially those related to higher cognitive functions. In this perspective, we suggest a theoretical framework for acquisition and control of hierarchical sequences. We bring together two hitherto unconnected streams of thought in this domain into one framework—the goal-directed and habitual axis on the one hand and the explicit and implicit sequencing paradigms on the other, with the help of model-based and model-free computational paradigms. We suggest that attentional engagement and disengagement allow the switching between these dichotomies. While goal-directed and habitual behaviors are related to performance of sequences, explicit and implicit paradigms relate to learning and acquisition of sequences. The unified computational framework proposes how the bidirectional flow in this hierarchy implements these two dichotomies. We discuss the neural correlates in light of this synthesis.

One aspect of applicability of our proposed framework could be skill learning. It is well known that skill learning proceeds from initially being slow, attentive and error-prone to finally being fast, automatic and error-free (Fitts and Posner, 1967). Thus, it appears that sequential skill learning starts being explicit and proceeds to be implicit from the point of view of attentional demands. At first sight, this seems to be at odds with the proposed unified framework here where the hierarchy seems to have been set up to proceed from implicit to explicit learning. However, the phase-wise progression of skill learning is consistent with the framework as per the following discussion.

It is pointed out that different aspects of skill are learned in parallel in different systems—while improvements in reaction time are mediated by implicit system, increasing knowledge of the sequential regularities accrues in the explicit system (Hikosaka et al., 1999; Bapi et al., 2000). The proposed unitary network is consistent with these parallel processes, the implicit processes operating from bottom-up and the explicit system in a top-down fashion. Key factor is the engagement and disengagement of attentional system as demarcated in Figure 1. One might wonder how this approach can be applied to research in non-human animals, where explicit mechanisms are difficult to be realized. Historically, while SRT research identifying implicit vs. explicit learning systems are largely based on human experiments, that of goal-directed and habitual research is based on animal experiments. The proposed framework is equally applicable for human and non-human participants. What is proposed here is that the lower-level system operates based on associative processes that allow the system to learn implicitly, respond reactively and the computations at this level are compatible with a model-free framework. On the other hand, the upper-level system is based on predictive processes that allow the system to prepare anticipatory responses that sometime cause errors. Error-evaluation while learning and error-monitoring during control are part of this system that learns using explicit processes, enables goal-directed control of actions and the computations at this level are compatible with a model-based framework. Level of attentional engagement distinguishes these two levels as shown in Figure 2. Of course, non-human animals can not give verbal reports of their knowledge. The explicit system in the case of pre-verbal infants and non-human animals needs to be understood in the lines of predictive systems that can elicit anticipatory, predictive responses and learn rules and transfer them to novel tasks (Marcus et al., 1999; Murphy et al., 2008).

Finally based on this theoretical proposal, we make predictions as to how implicit-to-explicit transition might happen in serial reaction time tasks when response-to-stimulus interval (RSI) is systematically manipulated. The mathematical formulation of such a unified mechanism is yet to be established, along with a formalization of the attentional window and its relation to RSI.

## Author contributions

TS, AS, and RB conceptualized the framework. TS and AS did literature review and wrote the introduction and review sections. RB added the modeling linkages. TS, AS, and RB all contributed to preparing and finalizing the manuscript.

## Funding

This work was partially supported by Department of Science and Technology (DST), under both Indo-Trento Program of Advance Research under Cognitive Neuroscience theme (No. INT/ITALY/ITPAR-III/Cog-P(6)/2013(C) dated 08-08-2013) as well as Indo-French CEFIPRA Grant for the project Basal Ganglia at Large (No. DST-INRIA 2013-02/Basal Ganglia dated 13-09-2014)—grants awarded to RB.

### Conflict of interest statement

The authors declare that the research was conducted in the absence of any commercial or financial relationships that could be construed as a potential conflict of interest.
